# Artificial intelligence in diagnosis of knee osteoarthritis and prediction of arthroplasty outcomes: a review

**DOI:** 10.1186/s42836-022-00118-7

**Published:** 2022-03-05

**Authors:** Lok Sze Lee, Ping Keung Chan, Chunyi Wen, Wing Chiu Fung, Amy Cheung, Vincent Wai Kwan Chan, Man Hong Cheung, Henry Fu, Chun Hoi Yan, Kwong Yuen Chiu

**Affiliations:** 1 Department of Orthopaedics and Traumatology, The University of Hong Kong, Hong Kong, China; 2 Department of Biomedical Engineering, The Hong Kong Polytechnic University, Hong Kong, China; 3 Department of Orthopaedics and Traumatology, Queen Mary Hospital, Hong Kong, China; 4 Department of Orthopaedics and Traumatology, Gleneagles Hospital Hong Kong, Hong Kong, China

**Keywords:** Artificial intelligence, Machine learning, Arthroplasty, Replacement, Total knee arthroplasty, Osteoarthritis

## Abstract

**Background:**

Artificial intelligence is an emerging technology with rapid growth and increasing applications in orthopaedics. This study aimed to summarize the existing evidence and recent developments of artificial intelligence in diagnosing knee osteoarthritis and predicting outcomes of total knee arthroplasty.

**Methods:**

PubMed and EMBASE databases were searched for articles published in peer-reviewed journals between January 1, 2010 and May 31, 2021. The terms included: ‘artificial intelligence’, ‘machine learning’, ‘knee’, ‘osteoarthritis’, and ‘arthroplasty’. We selected studies focusing on the use of AI in diagnosis of knee osteoarthritis, prediction of the need for total knee arthroplasty, and prediction of outcomes of total knee arthroplasty. Non-English language articles and articles with no English translation were excluded. A reviewer screened the articles for the relevance to the research questions and strength of evidence.

**Results:**

Machine learning models demonstrated promising results for automatic grading of knee radiographs and predicting the need for total knee arthroplasty. The artificial intelligence algorithms could predict postoperative outcomes regarding patient-reported outcome measures, patient satisfaction and short-term complications. Important weaknesses of current artificial intelligence algorithms included the lack of external validation, the limitations of inherent biases in clinical data, the requirement of large datasets in training, and significant research gaps in the literature.

**Conclusions:**

Artificial intelligence offers a promising solution to improve detection and management of knee osteoarthritis. Further research to overcome the weaknesses of machine learning models may enhance reliability and allow for future use in routine healthcare settings.

## Background

Total knee arthroplasty (TKA) is the only definitive surgical treatment for advanced knee osteoarthritis (KOA) [1–3]. Artificial intelligence (AI) and machine learning (ML) modeling is a new decision aid tool used in KOA diagnosis, patient selection, pre-TKA planning, prediction of disease progression, and estimation of treatment outcomes. The tool is improving with technological advancements and larger datasets but also requires extensive validation.

AI is a broad term referring to technologies that simulate human intelligence to automate tasks with high accuracy and precision. There are different methods to achieve this goal, such as designing algorithms with explicit rules and instructions or employing more “intelligent” algorithms such as those developed through machine learning. ML is a branch of AI involving algorithms that automatically “learn” from data, with incremental optimization and improvements in accuracy during the training process [2, 4]. Deep learning is a form of ML that does not require a labelled or structured dataset [4, 5]. For example, the use of artificial neural networks (utilizing the layers of increasing complexity and abstraction for information processing) to “learn” the important features of a model without human input [4].

AI can handle very large, complex datasets, and generate predictions to improve accuracy and efficiency of healthcare decisions, such as KOA and TKA [1]. ML algorithms have also been used to develop models to assist with pre-TKA planning and predict the value metrics of TKA, such as predicting implant size [6], reconstructing three-dimensional CT data of lower limb to facilitate robotic-assisted TKA [7], and assisting with component positioning and alignment [8]. ML potentially improves surgical precision and reduce the cost of manual labor. Regarding value metrics, ML methods have been used to predict the length of hospital stay, hospitalization charges, and discharge disposition. It impacts the economic burden of TKA and thus potentially affects decisions on payment models in healthcare settings [9–11].

This review aimed to summarize the existing evidence and highlight recent developments of AI and ML in diagnosis of KOA, prediction of the need for and outcomes of TKA.

## Materials and methods

We searched PubMed and EMBASE databases for articles published in peer-reviewed journals between January 1, 2010 and May 31, 2021. We searched for the following terms: ‘AI’, ‘machine learning’, ‘knee’, ‘osteoarthritis’, and ‘arthroplasty’. We selected studies focusing on the use of AI in diagnosis of KOA, predicting the need for TKA, and predicting outcomes of TKA. We excluded non-English language articles and the articles with no English translation. A reviewer screened the articles for the relevance to the research questions and strength of evidence.

## Results

The search produced 136 individual results, among which a total of 22 papers were included in the narrative synthesis following screening against inclusion/exclusion criteria (Table 1). Only one study was externally validated by testing the model using a dataset not used during model training to assess model performance and generalizability. The most commonly reported metric among the published articles was the area under the receiver operating characteristic curve (AUC), which evaluates the ability of an algorithm in discriminating between the individuals who experienced and those who did not experience the outcomes immediately after surgery and thereafter. AUC values ranged from 0.5 (indicating performance equal to a random predictor) to 1 (indicating a perfect predictor). Other reported metrics included sensitivity, specificity, Kappa coefficient (a measure of inter-rater reliability, where a value of 0 indicates no agreement while a value of 1 indicates perfect agreement), and positive and negative predictive values. The characteristics, performance, strengths, and weaknesses of AI algorithms are summarized in Table 2. AI algorithms used to predict the outcomes of TKA are shown in Table 3.

**Table 1:** Studies included in the scoping review

Area	First author	Year	Journal	Reference no.
OA diagnosis and TKA need	El-Galaly, A.	2020	Clinical Orthopaedics and Related Research	[12]
	Heisinger, S.	2020	Journal of Clinical Medicine	[13]
	Jafarzadeh, S.	2020	Osteoarthritis Cartilage	[14]
	Leung, K.	2020	Radiology	[15]
	Tolpadi, A.A.	2020	Scientific Reports	[16]
	Yi, P. H.	2020	Knee	[17]
	Norman, B.	2019	Journal of Digital Imaging	[18]
	Tiulpin, A.	2018	Scientific reports	[19]
Postoperative outcomes	Harris, A.	2021	The Journal of Arthroplasty	[20]
	Bonakdari, H.	2020	Computer Methods and Programs in Biomedicine	[21]
	Farooq, H.	2020	The Journal of Arthroplasty	[22]
	Hyer, J.M.	2020	Journal of the American College of Surgeons	[23]
	Ko, S.	2020	Knee Surgery, Sports Traumatology, Arthroscopy	[24]
	Kunze, K.	2020	The Journal of Arthroplasty	[25]
	Fontana, M. A.	2019	Clinical Orthopaedics and Related Research	[26]
	Harris, A.	2019	Clinical Orthopaedics and Related Research	[27]
	Huber, M.	2019	BMC Medical Informatics and Decision Making	[28]
	Lee, H. K.	2019	IEEE Journal of Biomedical and Health Informatics	[29]
	Aram, P.	2018	American Journal of Epidemiology	[30]
	Huang, Z.	2018	Transfusion	[31]
	Kluge, F.	2018	Gait Posture	[32]
	Van Onsem, S.	2016	The Journal of Arthroplasty	[33]

**Table 2:** A summary of reviewed studies on knee osteoarthritis diagnosis and knee arthroplasty prediction

Author (Year)	Journal	Prediction outcome	AI/ML algorithm(s)	Statistical performance	Strengths	Weaknesses	Clinical significance of study
Norman (2019) [18]	Journal of Digital Imaging	OA severity (KL grade)	DenseNet neural network architectures	Sensitivity & specificity: 84% & 86% (KL grades 0–1), 70% & 84% (KL grade 2), 69% & 97% (KL grade 3), 86% & 99% (KL grade 4).	Comparable sensitivity and specificity to manual KL grading and previous automatic systems employing different AI/ML algorithms	Training, validation and testing sets were selected from the same dataset. Misclassifications of KL grading typically occurred when there was hardware in the knee.	Provides additional data supporting the potential of AI in automatic assessment of OA radiological severity.
Tiulpin (2018) [19]	Scientific reports	OA severity (KL grade)	Deep Siamese CNN architecture	Average multi-class accuracy: 66.71%. AUC: 0.93. Kappa coefficient (agreement with expert annotations on test dataset): 0.83 (excellent). MSE value: 0.48.	Different datasets used for initial training and testing	Validation and testing sets were selected from the same dataset.	The provision of probability distributions for each KL grade prediction may assist clinicians in choosing KL grade in ambiguous cases.
Heisinger (2020) [13]	Journal of Clinical Medicine	Need for TKA	Artificial neural networks (ANNs) with linear, radial basis function and three-layer perceptron neural networks architectures	Total percentage of correctly predicted knees: 80%. Positive predictive value: 84%. Negative predictive value: 73%. Sensitivity: 41%. Specificity 30%.	First study to consider longitudinal change in symptomology (pain, function, quality of life) and radiographic structural change in a 4-year period prior to TKA	Training and testing sets were selected from the same dataset.	Future externally validated algorithms that can predict TKA need in advance using routinely available patient data could be highly useful for decisions for referral and triage in a primary care setting.
Leung (2020) [15]	Radiology	Need for TKA	Multitask deep learning model (ResNet34) trained with transfer learning	AUC: 0.87. Sensitivity: 83%. Specificity: 77%.	First study to directly predict TKA from knee radiographs using deep learning model	Limited data size (radiographs from 728 individuals in total) / Training and testing sets were selected from the same dataset.	TKA prediction models solely based on radiological data have limited clinical utility, although they may serve as a reference for future ML studies.
El-Galaly (2020) [12]	Clinical Orthopaedics and Related Research	Need for early revision TKA	LASSO regression, random forest classifier, gradient boosting model, neural network	AUCs: 0.57–0.60.	First study to predict early revision TKA (≤ 2 years of primary TKA) using preoperative patient data from arthroplasty registries / Temporal external validation was conducted (testing set selected from a separate hold-out year not included in training set).	Training and testing sets were selected from the same dataset.	Results from this study suggest that future models predicting early revision TKA may benefit from including more pre-operative information or predicting revision over a longer follow-up duration.

**Table 3:** A summary of reviewed studies on predicting postoperative outcomes of total knee arthroplasty

Author (Year)	Journal	Prediction outcome	AI/ML algorithm(s)	Statistical performance	Strengths	Weaknesses	Clinical significance of study
Huber (2019) [28]	BMC Medical Informatics and Decision Making	Postoperative improvement in PROMs	Extreme gradient boosting, multi-step adaptive elastic-net, random forest, neural net, Naïve Bayes, k-Nearest Neighbors	AUCs: 0.86 (VAS) & 0.70 (Q score).	Comparison of a wide variety of ML approaches in addition to regression methods	Training and testing sets were selected from the same dataset.	Identified important predictors for postoperative PROMs (e.g., preoperative VAS).
Harris (2021) [20]	The Journal of Arthroplasty	Postoperative 1-year achievement of MCID	LASSO regression, GBM, quadratic discriminant analysis	AUC: 0.76 (ADL), 0.72 (pain), 0.72 (symptoms), 0.71 (quality of life).	Provided sensitivity and specificity of various thresholds of predicted probability of failure to achieve MCID 1 year post-TKA.	Training and testing sets were selected from the same dataset.	Demonstrated potential for AI to predict patients most likely to benefit from TKA.
Kunze (2020) [25]	The Journal of Arthroplasty	Postoperative patient dissatisfaction	Stochastic gradient boosting, random forest, support vector machine, neural network, elastic-net penalized logistic regression	AUC: 0.66–0.79.	All five machine learning algorithms demonstrated superior predictive performance than the standard logistic regression model. Algorithm-identified predictors of postoperative patient dissatisfaction are consistent with previous systematic reviews.	Training and testing sets were selected from the same dataset.	Demonstrated potential for AI to predict patients most likely to experience postoperative dissatisfaction.
Farooq (2020) [22]	The Journal of Arthroplasty	Postoperative patient satisfaction	Stochastic gradient boosting	AUC: 0.81. Sensitivity: 73.0%. Specificity: 74.6%.	Demonstrated superior predictive performance than the binary logistic regression model.	Limited sample size (data from 897 cases) / Training and testing sets were selected from the same dataset.	Identified important predictors for postoperative patient satisfaction (e.g., age).
Harris (2019) [27]	Clinical Orthopaedics and Related Research	Postoperative 30-day complications and mortality	LASSO regression	AUC: 0.72 (cardiac complications), 0.69 (mortality), 0.60 (renal complications).	Different datasets were used for initial training and testing.	Training dataset (ACS-NSQIP) does not contain complete patient medical data (e.g., comorbidities), and includes patients from a limited number of hospitals.	Developed an externally validated model using routine clinical data as predictors and could therefore potentially be used to identify high-risk patients preoperatively.

### Diagnosis and predicting the need for TKA

Multiple machine learning models have been developed for radiological diagnosis and severity grading of KOA (based on the most widely used the Kellgren-Lawrence Classification System) (Table 2). Tiulpin et al. [19] developed an automatic grading model based on the Deep Siamese Convolutional Neural Network. The model was first trained using 18,376 knee radiographs from the Multicenter Osteoarthritis Study (a longitudinal, prospective, observational study of KOA in older Americans), and further tuned for hyperparameters using 2,957 KOA radiographs from the Osteoarthritis Initiative (a multicenter, longitudinal, prospective observational study of knee osteoarthritis), and finally tested on 5,960 randomly selected KOA radiographs from the Osteoarthritis Initiative that are unseen during the training process. The model achieved a kappa coefficient of 0.83 and an average multiclass accuracy of 67%, indicating excellent agreement (comparable to intra- and inter-rater reliability by arthroplasty surgeons) [34, 35]. The key benefit of this model is the provision of probability distributions for each Kellgren-Lawrence grade prediction. In clinical practice, the model may be used to select the closest Kellgren-Lawrence grade in ambiguous cases. Similarly, Norman et al. [18] used DenseNet neural network architectures to develop an automatic Kellgren-Lawrence grading model. Saliency maps revealed important radiographic features in algorithm’s decision-making, such as osteophytes and joint space narrowing. For detecting Kellgren-Lawrence grades, the sensitivity and specificity of the model were 69–86% and 84–99%, respectively. The kappa coefficient was 0.83, which was the same as the model proposed by Tiulpin et al. [19]. Most existing algorithms focus on the radiographic diagnosis of KOA or rely heavily on radiographic information as candidate predictors of TKA. This may be due to substantially increased imaging data availability following the recent creation of public datasets such as the Osteoarthritis Initiative.

In a recent study, Leung et al. [15] developed a deep learning model that directly predicted the need for TKA based on knee radiographs. This model demonstrated superior performance in predicting TKA than the conventional binary outcome models based on the Kellgren-Lawrence or Osteoarthritis Research Society International grades. The deep learning model used additional image-based information that might not be captured by simple numerical grading systems [36].

The discrepancies between radiologic and clinical severity of KOA have been widely reported [37–40]. Clinical diagnosis is typically made according to American College of Rheumatology criteria, taking into account patient age, symptoms, physical examination, and radiographic assessments [41]. The decision for surgery is driven primarily by symptom severity instead of radiological findings. Thus, the ML algorithms (automate Kellgren-Lawrence grading or predict TKA using imaging data alone) are limited in clinical decision-making. Nevertheless, the ML-based studies mentioned above offer insight to the development of radiograph-based prediction models using different machine learning approaches and may serve as a stepping stone to future studies that include additional clinical parameters, which may be more suitable for clinical decision-making support.

In 2020, Heisinger et al. [13] first designed an ML prediction model by investigating knee symptomatology (e.g., pain, function, and quality of life), Kellgren-Lawrence grading, and socioeconomic and demographic factors four years before TKA. The longitudinal analyses showed that significant worsening in knee symptomatology before TKA was the most important factor in decision making for TKA, compared to the radiographic progression of KOA. The artificial neural network can predict patients who may undergo TKA in the next two years with an accuracy of 80%, with a positive predictive value of 84%, and a negative predictive value of 73%.

El-Galaly et al. [12] were the first to attempt to develop a clinical ML algorithm to predict early revision TKA using preoperative data. The models were trained on the Danish Knee Arthroplasty Registry. Patient age, post-fracture osteoarthritis, and weight were statistically significant preoperative factors. Nevertheless, the authors were unable to develop a clinically useful model based on preoperative information [12]. Hence, further study is needed to identify clinically useful predictors of revision TKA.

### Predicting postoperative outcomes of TKA

The improvement following TKA is commonly assessed using the patient-reported outcome measures with or without accompanying “minimally clinically important improvement”, i.e., the minimum benefit assessed with the patient-reported outcome measures [42, 43]. Huber et al. [28] used ML algorithms to predict postoperative improvement in the patient-reported outcome measures. The models were trained and tested using the National Health Service data (130,945 observations), and the area under the receiver operating characteristic curve of the best performing models was approximately 0.86 (visual analogue scale) and 0.70 (Q score, i.e., sum of the Oxford Hip Score and Oxford Knee Score) for TKA. The results showed that preoperative visual analogue scale, Q score, and specific Q score dimensions were the most important predictors of postoperative patient-reported outcome measures [28]. Harris et al. [20] developed another model to predict post-TKA 1-year achievement of MCID and demonstrated fair discriminative ability for the prediction of some, but not all, PROMs included. Further development of similar machine learning algorithms for routine patient care could potentially assist postoperative outcome prediction.

AI can be used to predict post-TKA patient dissatisfaction. Kunze et al. [25] developed a random forest algorithm which demonstrated an AUC of 0.77 in identifying patients most likely to experience dissatisfaction. Farooq et al. [22] found that models built using ML achieved significantly higher AUC than using binary logistic regression on the same dataset (0.81 vs. 0.60). Given that a significant 20% of patients are dissatisfied following TKA and that existing statistical models cannot fully explain the reason for dissatisfaction [22], supervised machine learning models offer an alternative approach to automate the search for predictors of patient dissatisfaction.

The major complications of TKA are bleeding, thromboembolism, vascular injury, etc. [44] Many risk prediction calculators exist, such as the American College of Surgeons-National Surgical Quality Improvement Program universal surgical risk calculator and other arthroplasty-specific calculators [45, 46]. These conventional calculators have substantial weaknesses, such as poor accuracy, limited generalizability to external datasets, and preoperative use restrictions due to requiring intraoperative data as input variables [47, 48]. ML models offer an alternative approach to predict postoperative complications. Harris et al. [27] developed prediction models for 30-day mortality and major complications following elective arthroplasty. The models were trained on the American College of Surgeons National Surgical Quality Improvement data and externally validated using Veterans Affairs Surgical Quality Improvement Program data which had different patient demographics and clinical characteristics compared to the training data. The models showed acceptable performance in predicting mortality (AUC: 0.69) and cardiac complications (AUC: 0.72) (but not renal complications – AUC: 0.60) during external validation using the Veterans Affairs Surgical Quality Improvement Program data [27]. One important limitation of this study design is that the training dataset does not contain complete patient medical data (e.g., comorbidities) and only includes the patients from a small number of hospitals, limiting its generalizability [27]. Overall, ML has not been extensively applied in predicting post-TKA complications, and further efforts in model development with rigorous internal and external validation are warranted.

## Discussion

We find AI and ML models improve automatic grading of knee radiographs, patient selection for TKA, and predictin of postoperative outcomes of patient-reported outcome measures, patient satisfaction, and short-term complications. The weaknesses of current AI algorithms include the lack of external validation, inherent biases of clinical data, the need for large datasets for training, and significant research and regulatory gaps.

### Weaknesses of AI in arthroplasty

The current use of artificial intelligence algorithms has its limitations. First, accuracy and generalizability are key obstacles as very few models have been externally validated, and high AUC values do not necessarily translate to good clinical performance [26]. More rigorous external validation of prediction models is needed during algorithm development and testing, to ensure robustness and reliability before algorithms can be considered for routine clinical use. An important issue regarding generalizability lies in the fact that patient selection and postoperative outcomes are influenced by structure- and region-related confounders, such as institutional policies, hospital sites, and organizational culture [10]. For example, the threshold for booking TKA may differ between institutions depending on resource availability and hospital policy. Institutions may benefit from using region-specific machine learning algorithms for more accurate predictions.

Second, a practical disadvantage of machine learning models is the requirement of large datasets to train these models. These datasets often contain millions of unique data points and require hours or days of training, and additional datasets are needed to assess generalizability [49]. The increased availability of public datasets such as Multicenter Osteoarthritis Study and OAI could help overcome this obstacle and facilitate further research on machine learning in arthroplasty.

Third, a common concern surrounding the use of artificial intelligence is the “black-box” nature of machine learning models. Machine learning algorithms’ decision-making processes are opaque, using hidden layers and unknown connections between inputs and outputs, resulting in poor understanding and difficult scientific interpretation of how it generates predictions and recommendations [50]. Visualization of attention maps cannot directly provide information on these hidden relationships, and other efforts to increase the transparency of deep learning models are still ongoing [51]. Nevertheless, this poses more of a problem to scientific understanding rather than clinical application. By contrast, the reliance on data for model development is a key limitation of artificial intelligence in clinical use. Models developed are limited by the biases and limitations of current clinical data. Machine learning models are also “plastic”, i.e., changing when presented with new data [50], and the input parameters included in a machine learning algorithm, such as models predicting TKA need, may continuously change as new data becomes available to the model.

Finally, significant research and regulatory gaps exist, given the novel nature of this technology. There is a paucity of literature on the use of machine learning algorithms to predict the need for arthroplasty, and current machine learning models are unable to predict the long-term outcomes of TKA. ML models are limited by the biases of current clinical data, and future implementation of these algorithms into routine hospital care will also come with regulatory concerns of algorithm quality control, security issues and adversarial attacks.

## Conclusions

KOA is an important public health problem worldwide. AI offers a promising solution to detect KOA and improve pre-TKA planning. Further research is needed to overcome the limitations of ML models and ensure reliability for future use in routine healthcare settings.

## Data Availability

All data generated or analysed during this study are included in this published article.

## Abbreviations

ACS-NSQIP: American College of Surgeons-National Surgical Quality Improvement Program
AUC: Area under the receiver operating characteristic curve
KL: Kellgren & Lawrence
LOS: Length of stay
ML: Machine learning
KOA: Knee osteoarthritis
OAI: Osteoarthritis Initiative
TKA: Total knee arthroplasty
VASQIP: Veterans Affairs Surgical Quality Improvement Program
